# Reviewed article: Feitosa YS, Cabral BVB, Lima GS, Gomes EB, Sousa GJB, Moreira TMM, Pereira MLD. Psychosocial risks and work ability among health workers throughout the COVID-19 pandemic: a longitudinal follow-up study, Brazil, 2020-2023. Epidemiol Serv Saude. 2026:35;e20240798

**DOI:** 10.1590/S2237-96222026v35e20240798.b

**Published:** 2026-03-09

**Authors:** Marilia Mastrocolla de Almeida Cardoso

**Affiliations:** 1 Ministério da Saúde, Brasília, DF, Brazil

## First round comments

O documento necessita de muitos ajustes conforme orientação dos revisores. Acrescento as observações a seguir:

Introdução

Alguns parágrafos da introdução necessitam de referência, como “Os riscos psicossociais relacionados ao trabalho resultam da interação entre o ambiente de trabalho e os fatores humanos, como capacidades e expectativas, podendo influenciar a saúde, desempenho e a capacidade para o trabalho. A pandemia de COVID-19 gerou alterações nas condições de trabalho dos profissionais de saúde sendo necessário acompanhar os efeitos da pandemia nos riscos psicossociais e capacidade para o trabalho nesta população. “Se a proposta do texto é comparar grupo de profissionais da saúde com profissionais de outras áreas, será importante apresentar dados sobre o adoecimentos em outras áreas para poder justificar a comparação. O adoecimento de profissionais de saúde por si só no período da pandemia, já é algo bastante estudado. Por que o grupo optou por essa comparação? Quais foram as hipóteses que nortearam a motivação do estudo?

Método

Como foi calculada a amostra? Por que há essa diferença de participantes entre os dois grupos? Como foi trabalhada essa diferença no tamanho amostral entre grupos?

Descrever com mais detalhes o que seriam os anúncios na imprensa local (o estudo é nacional), mídias sociais (pessoais, institucionais?) e convites enviados por e-mail (especificamente para quais e-mails? Quais critérios de seleção dos e-mails?). Quais limitações existiram nesse processo de contato? O que seria “setor econômico” ? recomendo especificar

Falta pontuação neste trecho “...perdeu emprego na pandemia questões sobre a pandemia COVID-19. “ E o que seriam questões sobre a pandemia?

Resultados

- Houve grande perda de participantes ao logo do estudo. Como esta questão foi trabalhada?

Discussão

A discussão aborda somente as questões relacionadas aos profissionais de saúde. No entanto, existem resultados que aparecem com maior frequência para os outros profissionais.

Embora o estudo compare dois grupos distintos, tanto na introdução, como na discussão, abordam somente

dados sobre os profissionais de saúde.

Recommendation: Major revision

## Second round comments

Prezados autores, ainda existem alguns ajustes que necessitam de atenção:

1) Excluir estado no titulo das ilustrações, deixar somente a cidade.

2) No titulo da tabela, colocar o ano da pesquisa e não da analise/escrita.

3) Inserir o separador de milhar em todos os numerais, além de ajustar a pontuação. Separar intervalos por ponto e vírgula, e com espaço entre a pontuação e numeral subsequente: “(IC95% 1,14; 2,23)”. Caso ambas as medidas sejam apresentadas entre parênteses, incluir ponto e vírgula para separar as medidas: “(RP 1,52; IC95% 1,14; 2,23)”.

4) Figura 2- essa categorização “não saúde” poderia ser simplesmente outros. Rever o título e ajustar conforme as normas da revista.

5) Rever títulos, pois não está adequado- “Os dados estão apresentados em frequência relativa (%) e intervalos de confiança de 95% (IC 95%).” Seria simplesmente colocar frequência e intervalos de confiança de 95% (IC 95%) dos aspectos...

6) Rever as orientações dadas para a ordem das informações na discussão: O primeiro parágrafo da discussãomdeve resumir os achados, interpretando-os e sem repetir números nem citar ilustrações ou referências: não informar o que fez, interpretar o que encontrou. Apresentar as limitações do estudo, levando em consideração fontes potenciais de viés ou imprecisão no segundo parágrafo. Após esclarecer as limitações da pesquisa, o texto da discussão deve ser guiado pelos seus achados. Interpretar cada resultado relevante e discuti-lo à luz da literatura. Esgotar o tema abordado e evitar retomá-lo adiante no texto de modo circular e redundante. Evitar parágrafos sem conexão com a literatura ou que somente a revisam, sem discussão com os resultados. Assegurar que a discussão faz a ponte entre os achados e pesquisas compatíveis - com delineamento, amostragem e demais aspectos metodológicos comparáveis ao que foi realizado na pesquisa -, ou evidenciar as discrepâncias; caracterizar o estudo citado (delineamento, pessoa, tempo e lugar) de modo a esclarecer semelhanças e diferenças pertinentes a tal confrontação. O último parágrafo (curto e conciso) deve apresentar a conclusão da pesquisa com base do que se pode afirmar a partir dos resultados.

Recommendation: Minor revision

## Third round comments

Prezados autores, seguem os últimos ajustes de formatação:

1) A versão enviada precisa seguir as normas da revista para formatação das citação de referência no texto: As referências devem ser citadas em sistema numérico, segundo a ordem de citação no texto, com os números entre parênteses, imediatamente após a passagem do texto em que é feita a citação, e antes da pontuação do texto, separados entre si por vírgulas; se números sequenciais, separados por hífen, enumerando apenas a primeira e a última referência do intervalo sequencial de citação; exemplo: (7,10-16).

2) A solicitação de inserção do separador de milhar em todos os numerais, se refere exclusivamente aos dados numéricos de resultados. Não deve ser inserido nos anos, (2.020 errado), deve manter 2020.

Recommendation: Minor revision

